# Supplementary material for: Purple sulfur bacteria fix N2 via molybdenum-nitrogenase in a low molybdenum Proterozoic ocean analogue
Source: Nat Commun. 2021 Aug 6;12:4774. doi: 10.1038/s41467-021-25000-z (PMC8346585; doi:10.1038/s41467-021-25000-z)
Supplement: Supplementary file 1 — Supplementary Information [file 41467_2021_25000_MOESM1_ESM.pdf]

# Supplementary Information

# Purple sulfur bacteria fix $N_2$ via molybdenum-nitrogenase in a low molybdenum Proterozoic ocean analogue

Miriam Philippi[1], Katharina Kitzinger[1]*, Jasmine S. Berg[2], Bernhard Tschitschko[1], Abiel T. Kidane[1], Sten Littmann[1], Hannah K. Marchant[1], Nicola Storelli[3], Lenny H. E. Winkel[2,4], Carsten J. Schubert[2,5], Wiebke Mohr[1], Marcel M. M. Kuypers[1]

[1]Department of Biogeochemistry, Max Planck Institute for Marine Microbiology, Bremen, Germany

[2]Department of Environmental Systems Science, ETH-Zurich, Zurich, Switzerland

[3]Laboratory of Applied Microbiology, Department of Environment, Constructions and Design, University of Applied Sciences of Southern Switzerland (SUPSI), Bellinzona, Switzerland

[4]Eawag, Swiss Federal Institute of Aquatic Science and Technology, Dübendorf, Switzerland

[5]Eawag, Swiss Federal Institute of Aquatic Science and Technology, Kastanienbaum, Switzerland

*For correspondence. Email kkitzing@mpi-bremen.de

## Supplementary Note S1: Genetic potential for $N_2$ fixation in the Lake Cadagno *Chromatium okenii* population

In addition to the three structural genes encoding for the MoFe nitrogenase enzyme, *nifH* (Figure 2), *nifD* (Figure S2) and *nifK* (Figure S3), we identified three other genes involved in $N_2$ fixation in the *Chromatium okenii* MAG (MAG 8): the Nif-specific regulatory protein gene *nifA*, the homocitrate synthase gene *nifV* and the pyruvate-flavodoxin oxidoreductase gene *nifJ*. The previously published *C. okenii* MAGs from Lake Cadagno (NCBI: SAMN08142835[1]; IMG genome ID: 2700988602[2]) encode additional $N_2$ fixation genes, including the nitrogenase molybdenum-cofactor synthesis protein gene *nifE*, the Mo donor protein gene *nifQ*, the $N_2$ fixation protein genes *nifX*, *nifB*, *nifT*, *nifZ* and *nifW*, as well as the regulator gene *nifL*. Taken together, the Lake Cadagno *C. okenii* population encodes for almost all genes regarded as essential for $N_2$ fixation, with the exception of *nifN*[3]. The *C. okenii* MAGs from Lake Cadagno represent incomplete genomes, as such, these organisms might actually encode for additional $N_2$ fixation genes currently not represented in the MAGs. In conclusion, the plethora of $N_2$ fixation genes found in the genome of the Lake Cadagno *C. okenii* population strongly supports the measured $N_2$ fixation activity of this microorganism (Figure 4).

## Supplementary Note S2: Other potential $N_2$-fixers in Lake Cadagno

PSB were shown to be key $N_2$-fixers in the chemocline of Lake Cadagno in 2018. Yet, also some non-targeted cells were found to be enriched in $^{15}N$ during nanoSIMS analysis (Figure S3). These cells most likely belonged to other potential $N_2$-fixers which were identified in the metagenome, some of which even showed active transcription of *nif* genes in the metatranscriptomes (Figures 2, S1 and Table S3). *Thiocystis*, Afipia or Desulfobacterota are possible $N_2$-fixers in Lake Cadagno, as well as *C. phaeobacteroides,* for which active $N_2$ fixation has previously been shown in this system[4]. Although some of these populations might have been present and active in the investigated depth, their contribution to the bulk $N_2$ fixation rates was likely low due to their low contribution to bulk biomass. Alternatively, the additionally observed enriched cells could have also developed from cross-feeding of exudates from highly enriched PSB[5].

# Supplementary Tables

**Table S1: Lake Cadagno sulfide and nutrient concentrations.** Concentrations of sulfide ($H_2S$), ammonium ($NH_4^+$), nitrate plus nitrite ($NO_x$), phosphate ($PO_4^{3-}$), reactive silica (Si) and molybdenum (Mo) are shown for the corrected depths.

| **Depth** (m) | **$H_2S$** ($\mu$mol $L^{-1}$) | **$NH_4^+$** ($\mu$mol $L^{-1}$) | **$NO_x$** ($\mu$mol $L^{-1}$) | **$PO_4^{3-}$** ($\mu$mol $L^{-1}$) | **Si** ($\mu$mol $L^{-1}$) | **Mo** (nmol $L^{-1}$) |
|---|---|---|---|---|---|---|
| 12.3 | 0.00 | 0.16 | 0.13 | 0.01 | 101.92 | 9.25 |
| 12.5 | 0.00 | 0.05 | 0.06 | 0.00 | 106.82 | 8.48 |
| 12.8 | 0.07 | 0.45 | 0.05 | 0.00 | 109.48 | 7.63 |
| 12.8 | 0.37 | 0.88 | 0.06 | 0.02 | 111.69 | 7.14 |
| 13.5 | 2.21 | 2.19 | 0.06 | 0.00 | 106.38 | 6.48 |
| 13.6 | 2.01 | 2.78 | 0.04 | 0.00 | 106.28 | 6.42 |
| 13.7 | 0.30 | 0.25 | 0.05 | 0.00 | 108.22 | 7.68 |
| 13.7 | 0.45 | 0.57 | 0.03 | 0.01 | 109.12 | 7.15 |
| 14.0 | 1.04 | 4.36 | 0.03 | 0.00 | 107.56 | 6.06 |
| 14.3 | 1.19 | 4.96 | 0.04 | 0.00 | 101.19 | 6.02 |
| 14.7 | 1.19 | 13.87 | 0.06 | 0.00 | 106.31 | 5.48 |
| 14.8 | 18.96 | 17.95 | 0.05 | 0.00 | 106.00 | 5.23 |
| 15.1 | 35.32 | 19.12 | 0.05 | 0.00 | 109.50 | 5.10 |
| 15.3 | 63.95 | 15.37 | 0.05 | 0.00 | 93.29 | 3.90 |
| 15.5 | 81.05 | 21.15 | 0.07 | 0.00 | 110.80 | 5.01 |
| 15.8 | 56.52 | 23.12 | 0.07 | 0.01 | 107.43 | 5.04 |
| 16.0 | 30.49 | 25.73 | 0.09 | 0.00 | 111.35 | 4.98 |
| 16.5 | 55.03 | 26.44 | 0.08 | 0.00 | 119.70 | 4.71 |
| 16.7 | 35.69 | 28.27 | 0.10 | 0.01 | 114.27 | 4.80 |
| 17.0 | 66.93 | 30.41 | 1.37 | 0.01 | 118.24 | 5.05 |

**Table S2: Natural abundance $\delta^{15}N$ values of in situ biomass as determined from measurements with an elemental analyzer coupled to a continuous-flow isotope ratio mass spectrometer.** Note the additional replicates, sampled on the $29^{th}$ of August, which are not included in the main text and figures.

| Sample | Sampling date | $\delta^{15}N$ (‰) |
|---|---|---|
| 13.7 m, replicate 1 | 2018/08/28 | 1.7 |
| 13.7 m, replicate 2 | 2018/08/28 | 1.7 |
| 14 m, replicate 1 | 2018/08/28 | 2.6 |
| 14 m, replicate 2 | 2018/08/28 | 1.8 |
| 15.5 m | 2018/08/28 | -2.1 |
| 13.6 m, replicate 1 | 2018/08/29 | 0.9 |
| 13.6 m, replicate 2 | 2018/08/29 | 2.0 |
| 15.7 m, replicate 1 | 2018/08/29 | 0.6 |
| 15.7 m, replicate 2 | 2018/08/29 | -0.3 |

**Table S3: Transcription of *nif* genes identified in the metagenome dataset.** The number of metatranscriptome reads, from the three different incubation depths are shown, which mapped to the *nif* genes found in the metagenome dataset. Only *nif* genes to which at least one transcriptome read mapped are shown.

| Bin | Gene | 13.7 m | 14 m | 15.5 m |
|---|---|---|---|---|
| MAG 4 | *nifD* | 0 | 1 | 0 |
| MAG 4 | *nifK* | 0 | 1 | 0 |
| MAG 5 | *nifD* | 0 | 2 | 0 |
| MAG 5 | *nifK* | 0 | 0 | 2 |
| MAG 7 | *nifK* | 0 | 3 | 0 |
| MAG 7 | *nifD* | 0 | 4 | 0 |
| MAG 7 | *nifH* | 0 | 1 | 0 |
| MAG 8 | *nifH* | 1 | 4 | 1 |
| MAG 8 | *nifD* | 2 | 5 | 0 |
| MAG 8 | *nifK* | 3 | 11 | 0 |
| MAG 10 | *nifK* | 0 | 1 | 0 |
| MAG 11 | *nifD* | 0 | 0 | 1 |
| MAG 13 | *nifH* | 1 | 0 | 0 |
| MAG 15 | *nifK* | 1 | 0 | 0 |
| MAG 34 | *nifH* | 1 | 0 | 0 |
| MAG 34 | *nifD* | 0 | 0 | 1 |
| unbinned | *nifK* | 0 | 1 | 0 |
| unbinned | *nifK* | 0 | 1 | 0 |
| unbinned | *nifK* | 1 | 0 | 0 |

**Table S4: Metatranscriptome sequencing overview**

| | 15.5 m | 14 m | 13.7 m |
|---|---|---|---|
| **Raw reads:** | 44,141,708 | 32,511,200 | 34,206,034 |
| **Trimmed reads:** | 43,967,442 | 32,384,456 | 34,072,142 |
| **Merged reads:** | 18,628,265 | 13,344,041 | 13,649,685 |
| **Average read length of merged reads (bp):** | 278 | 296 | 306 |
| **rRNA reads:** | 18,463,365 (99.11%) | 13,214,507 (99.02%) | 13,553,354 (99.29%) |
| **mRNA reads:** | 164,900 (0.88%) | 129,534 (0.97%) | 96,331 (0.70%) |

**Table S5: Analysis of a replicate incubation experiment performed on the following day (29th of August 2018).** Incubation experiments on the 29th of August were performed as described for the previous day using chemocline samples from 13.5 m water depth. The replicate chosen for single cell analysis with nanoSIMS is highlighted in bold. For the average bulk $N_2$ fixation rate, the standard deviation is shown. For *C. okenii* abundance, average cellular rate and contribution to bulk $N_2$ fixation, the respective standard errors are shown. For calculations of the cellular rates, the average biovolume of the *C. okenii* cells from the previous day was used, as cells analyzed in the sample from the 29th were largely overlapping and no accurate size determination was possible. No FISH or DAPI staining was performed and *C. okenii* cells were identified based on morphology.

| | |
|---|---|
| Bulk $N_2$ fixation rates of the individual replicates (nmol N $L^{-1}$ $d^{-1}$) | 41.35<br>118.60<br>**133.17** |
| Average bulk $N_2$ fixation rate (nmol N $L^{-1}$ $d^{-1}$) | 97.7<br>± 49.34 |
| Abundance of *C. okenii* (cells $L^{-1}$) | $4.27 \times 10^7$<br>$\pm 0.32 \times 10^7$ |
| Number of *C. okenii* cells analyzed with nanoSIMS | 25 |
| Average cellular *C. okenii* $N_2$ fixation rate (fmol N cell$^{-1}$ $d^{-1}$) | 1.9<br>± 0.29 |
| Contribution of *C. okenii* to the average bulk $N_2$ fixation rate (%) | 83.19<br>± 44.27 |

**Table S6: Metagenome assembly statistics**

| | |
|---|---|
| Number of scaffolds: | 247,183 |
| Total scaffold length (bp): | 638,673,456 |
| Scaffolds >1000bp: | 548,793,469 |
| Max. scaffold length: | 1,184,040 |
| N50: | 6,964 |
| L50: | 13,765 |

**Table S7: FISH probes and used formamide concentrations**

| Probe | Target | Sequence | Formamide | Reference |
|---|---|---|---|---|
| **Apur453** | *Lamprocystis purpurea* (DSM 4197) | TCGCCCAGGGTATTATCCCAAACGAC | 40% | Tonolla et al.[6] |
| **Cmok453** | *Chromatium okenii* (DSM 169) | AGCCGATGGGTATTAACCACCAGGTT | 30% | Tonolla et al.[6] |
| **Laro453** | *Lamprocystis roseopersicina* (DSM229) | CATTCCAGGGTATTAACCCAAAATGC | 30% | Tonolla et al.[6] |
| **S453D** | *Lamprocystis* spp. | CAGCCCAGGGTATTAACCCAAGCCGC | 40% | Tonolla et al.[6] |
| **Thiosyn459**<br>Helper1<br>Helper2<br>Helper3<br>Helper4<br>Competitor1<br>Competitor2 | *Thiodictyon syntrophicum* Cad16 | **TCAACCCTCATGGGTATTAACCAC (Thiosyn459)**<br>GCTTTACAACCCGCAGGCCTTCTT (Helper1)<br>AAGGCGTTCTTCCCCACTGAAAGT (Helper2)<br>CCGGTGCTTCTTCTGTGGGTAACG (Helper3)<br>GCGGCTGCTGGCACGGAGTTAG (Helper4)<br>TCAACCCTCATGGGTATTAGCCAC (Competitor1)<br>TCATCCCCCACGGGTATTAACCAC (Competitor2) | 35% | this study |

# Supplementary Figures

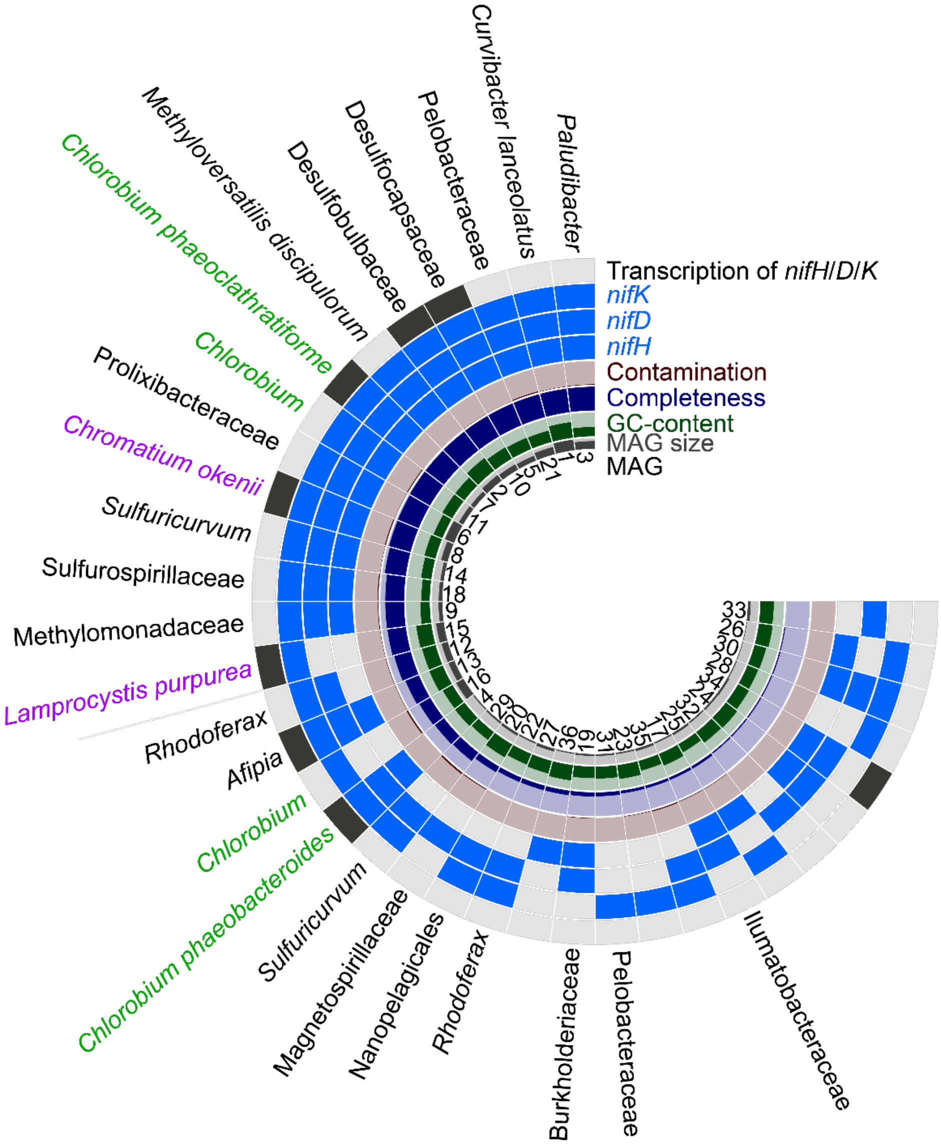


**Figure S1. Overview of nitrogenase gene-containing MAGs obtained from Lake Cadagno metagenomes (2014).** MAGs are sorted according to completeness, MAG taxonomy was inferred from GTDB-Tk classification (no taxonomy indicates no classification to known taxa due to low completeness and thus lack of marker genes of the MAGs). PSB and GSB are indicated in purple and green, respectively. Information depicted in circles from inner to outer circles: MAG ID number, MAG size, GC content, completeness and contamination. Completeness and contamination are shown from 0 to 100% and were obtained from CheckM analyses. Note that due to low contamination values, the bars are largely not visible. *nifH, nifD* and *nifK* circles indicate presence of the respective nitrogenase gene subunit in the MAGs. Outermost circle indicates whether nitrogenase transcription (any of the three genes) was detected in the 2018 metatranscriptomes.

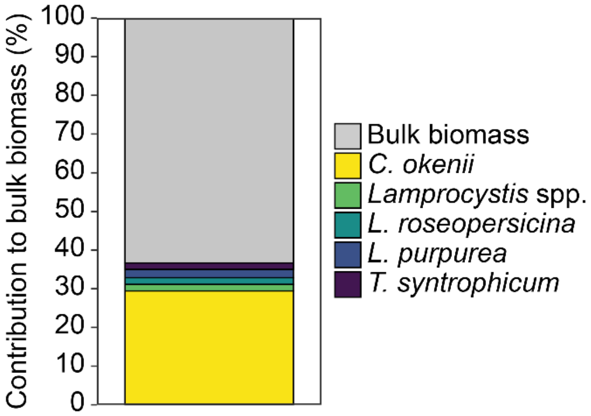


**Figure S2. Contribution of PSB populations to bulk carbon biomass at 13.7 m depth.**

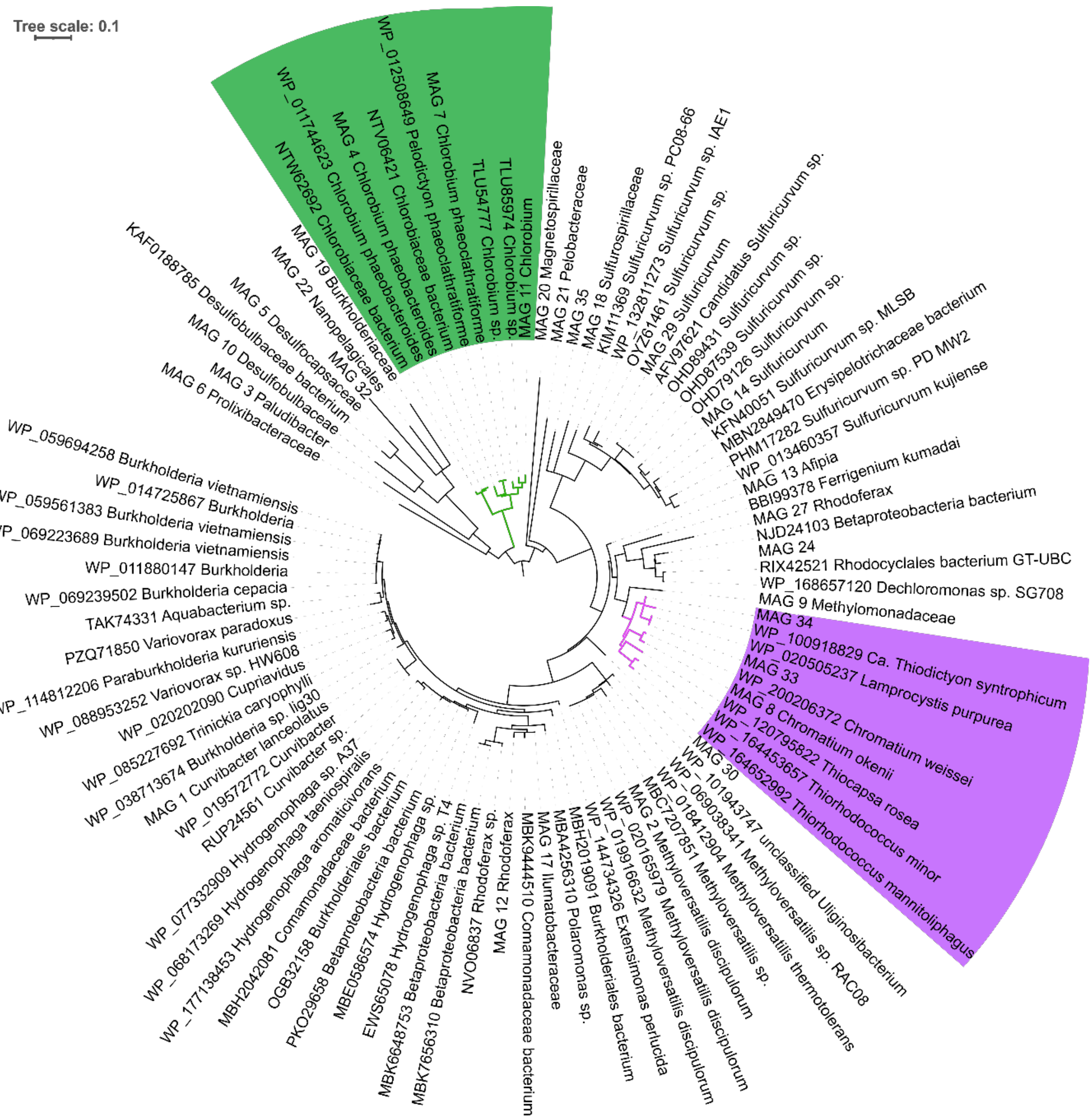


**Figure S3: NifD phylogenetic tree.** The tree includes all NifD amino acid sequences found within a nitrogenase gene-containing MAG and reference sequences. The accession numbers of the reference sequences are shown together with the NCBI taxonomy. MAG taxonomy was inferred from GTDB-Tk classification. The lowest taxonomic rank assigned is shown, with no taxonomic information indicating that classification based on GTDB-Tk was not possible due to a lack of sufficient marker genes.

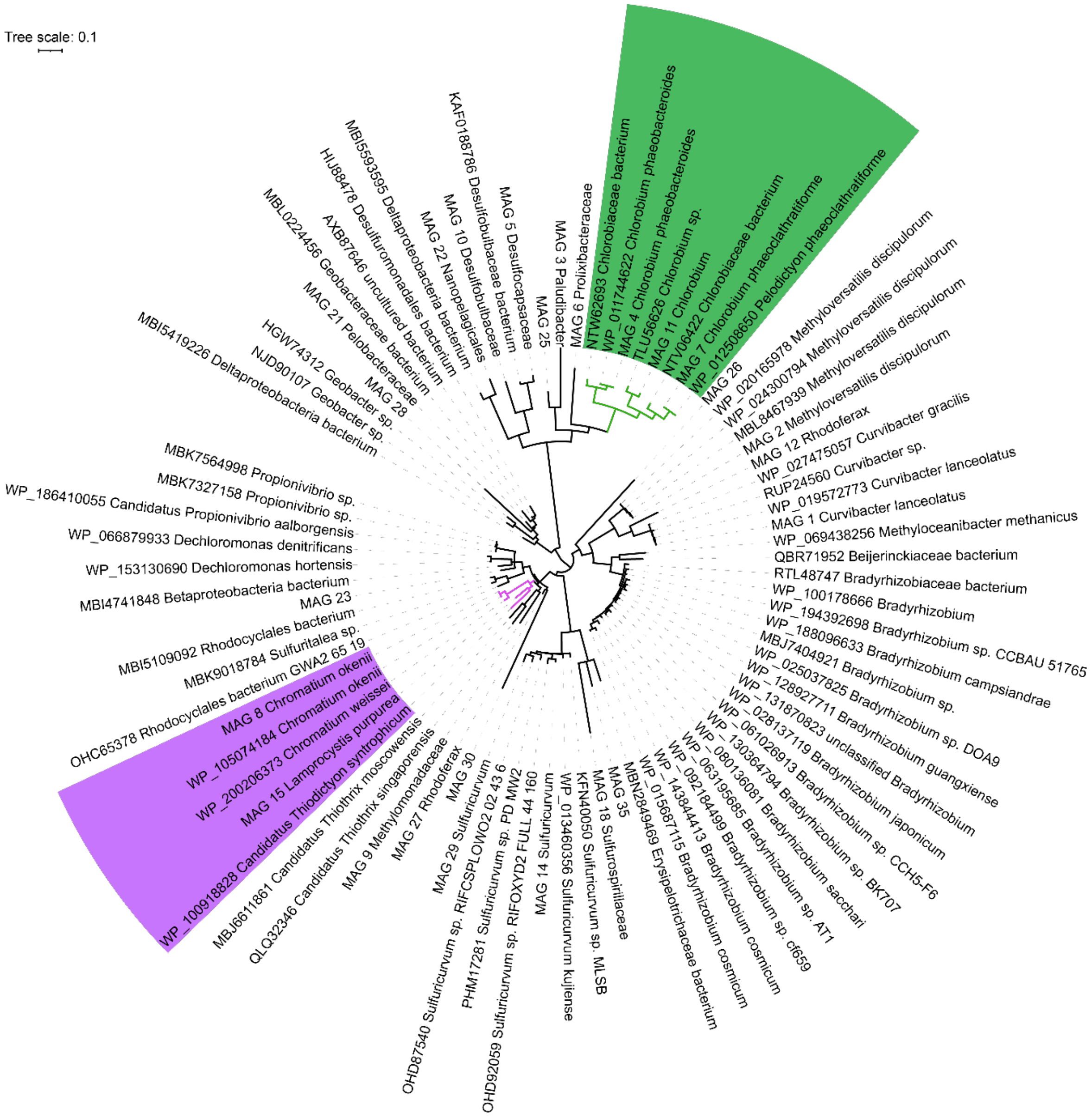


**Figure S4: NifK phylogenetic tree.** The tree includes all NifK amino acid sequences found within a nitrogenase gene-containing MAG, except the sequences of MAG 13, MAG 16 and MAG 31, as these sequences were too short for reliable tree reconstruction. The accession numbers of the reference sequences are shown together with the NCBI taxonomy. MAG taxonomy was inferred from GTDB-Tk classification. The lowest taxonomic rank assigned is shown, with no taxonomic information indicating that classification based on GTDB-Tk was not possible due to a lack of sufficient marker genes

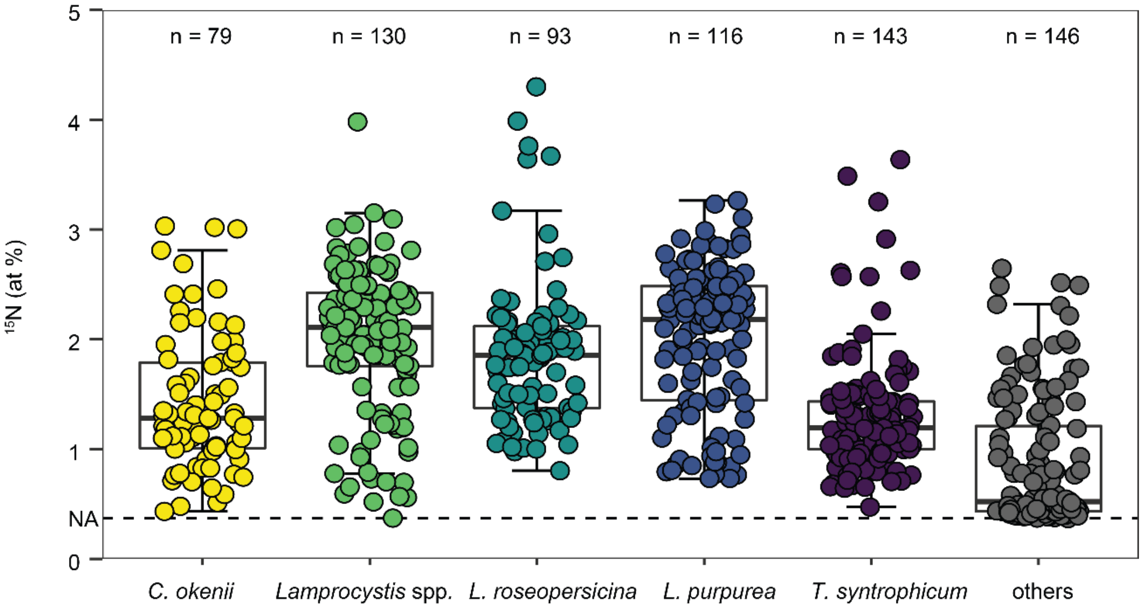


**Figure S5. Single cell $^{15}$N enrichment determined by nanoSIMS.** $^{15}$N atom% enrichment of individual PSB populations and other cells at 13.7 m depth. The number of cells analyzed per population (n) is shown above each box plot. The natural abundance $^{15}$N atom%, as determined for the respective water depth using an Elemental Analyzer (0.37%), is depicted as dashed line. Boxplots depict the 25–75% quantile range, with the center line depicting the median (50% quantile); whiskers encompass data points within 1.5× the interquartile range.

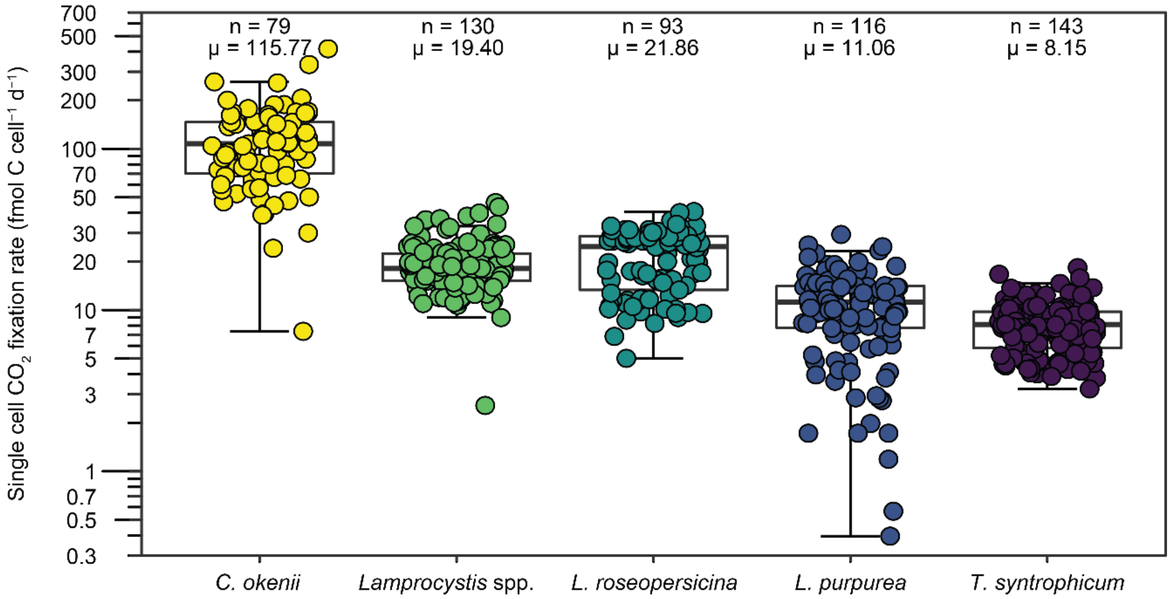


**Figure S6. Single cell $CO_2$ fixation activity determined by nanoSIMS.** Single cell $CO_2$ fixation rates of PSB populations at 13.7 m depth. The number of cells analyzed per population is shown (n) above each box plot together with the mean rate (μ). Boxplots depict the 25–75% quantile range, with the center line depicting the median (50% quantile); whiskers encompass data points within 1.5× the interquartile range. Y-axis is in log scale.

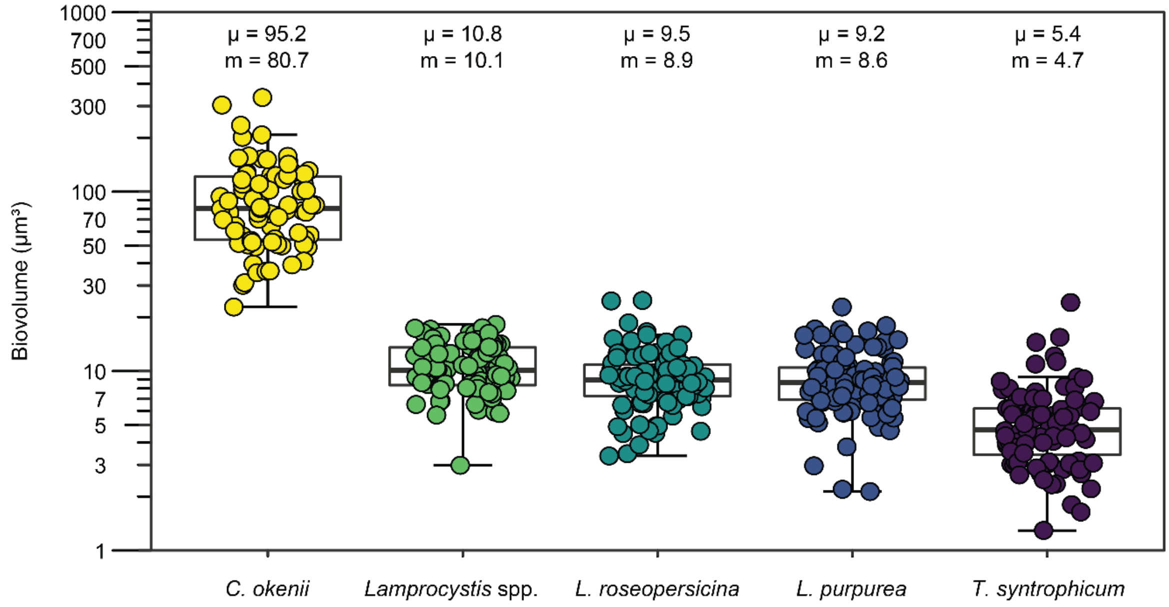


**Figure S7. Single cell biovolumes of the investigated PSB populations.** Biovolumes for cluster-forming PSB populations (*T. syntrophicum, L. roseopersicina, Lamprocystis sp., L. purpurea*) were calculated from each 100 cells using the measured cell dimensions in epifluorescence images. Biovolume of the non-cluster forming *C. okenii* cells were calculated from cell (ROI) dimensions obtained from nanoSIMS data. Boxplots depict the 25–75% quantile range, with the center line depicting the median (50% quantile); whiskers encompass data points within 1.5× the interquartile range. Mean (µ) and median (m) values are shown above the box plots. Y-axis is in log scale.

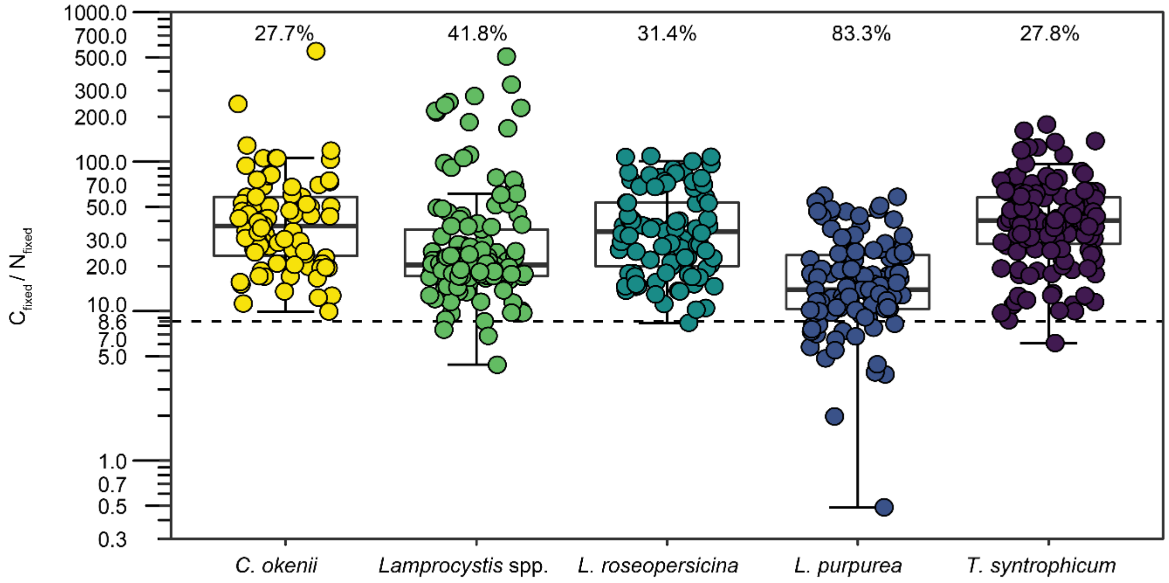


**Figure S8. C/N fixation ratios of PSB.** Ratios of fixed C to fixed N of individual PSB populations at 13.7 m depth. The average fraction of autotrophic N-demand (based on the per cell $CO_2$ fixation rate and bulk C/N ratios) met by $N_2$ fixation is shown in percent above each box plot. The bulk C/N ratio of the biomass at 13.7 m depth, as determined for the natural abundance control sample using an Elemental Analyzer (8.6), is depicted as a dashed line. We analyzed 79, 130, 93, 116 and 143 individual PSB cells for *C. okenii, Lamprocystis spp., L. roseopersicina, L. purpurea and T. syntrophicum* populations, respectively. Boxplots depict the 25–75% quantile range, with the center line depicting the median (50% quantile); whiskers encompass data points within 1.5× the interquartile range. Y-axis is in log scale.

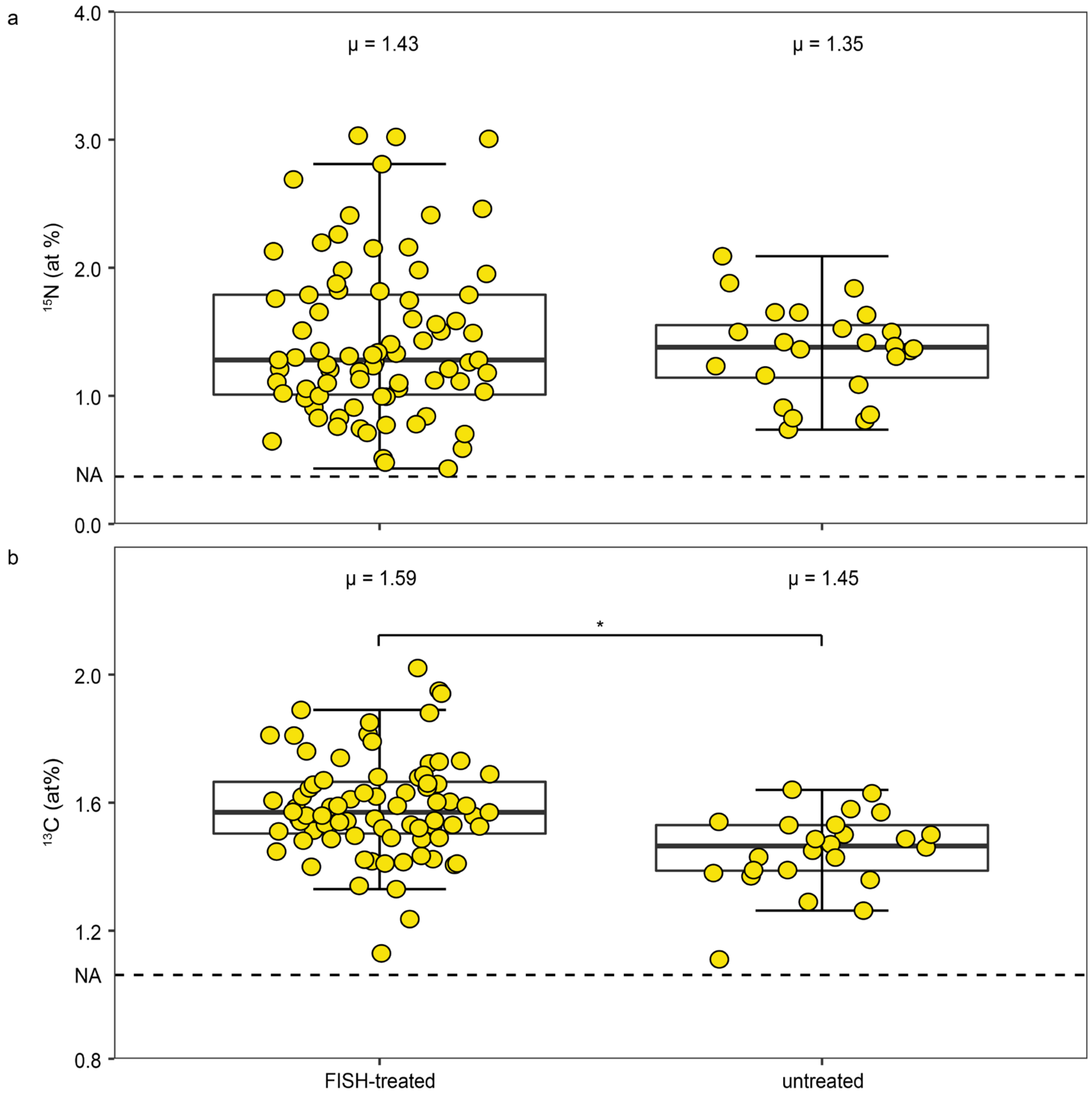


**Figure S9: Comparison of $^{15}$N and $^{13}$C enrichment of FISH-treated and untreated *C. okenii* cells.** To investigate the potential dilution effect of cellular isotope enrichment through FISH, we analyzed 24 *C. okenii* cells that did not undergo FISH and DAPI staining (untreated), in the same replicate sample that was used for all other nanoSIMS analyses and compared them to FISH-treated and DAPI stained *C. okenii*. The $^{15}$N at% (a) and the $^{13}$C at% (b) of the analyzed cells is shown. The average enrichment (μ) is indicated above each box. The natural abundance atom% (NA), as determined for the respective water depth using an Elemental Analyzer, is depicted as dashed line. Boxplots depict the 25–75% quantile range, with the center line depicting the median (50% quantile); whiskers encompass data points within 1.5× the interquartile range. Mean $^{13}$C at% enrichment significantly differed between FISH-treated and untreated cells, with lower enrichment in untreated cells (two sided, two-sample Wilcoxon test, $W = 1463.5$, p-value = $5.9 \times 10^{-5}$), as indicated by the asterisk.

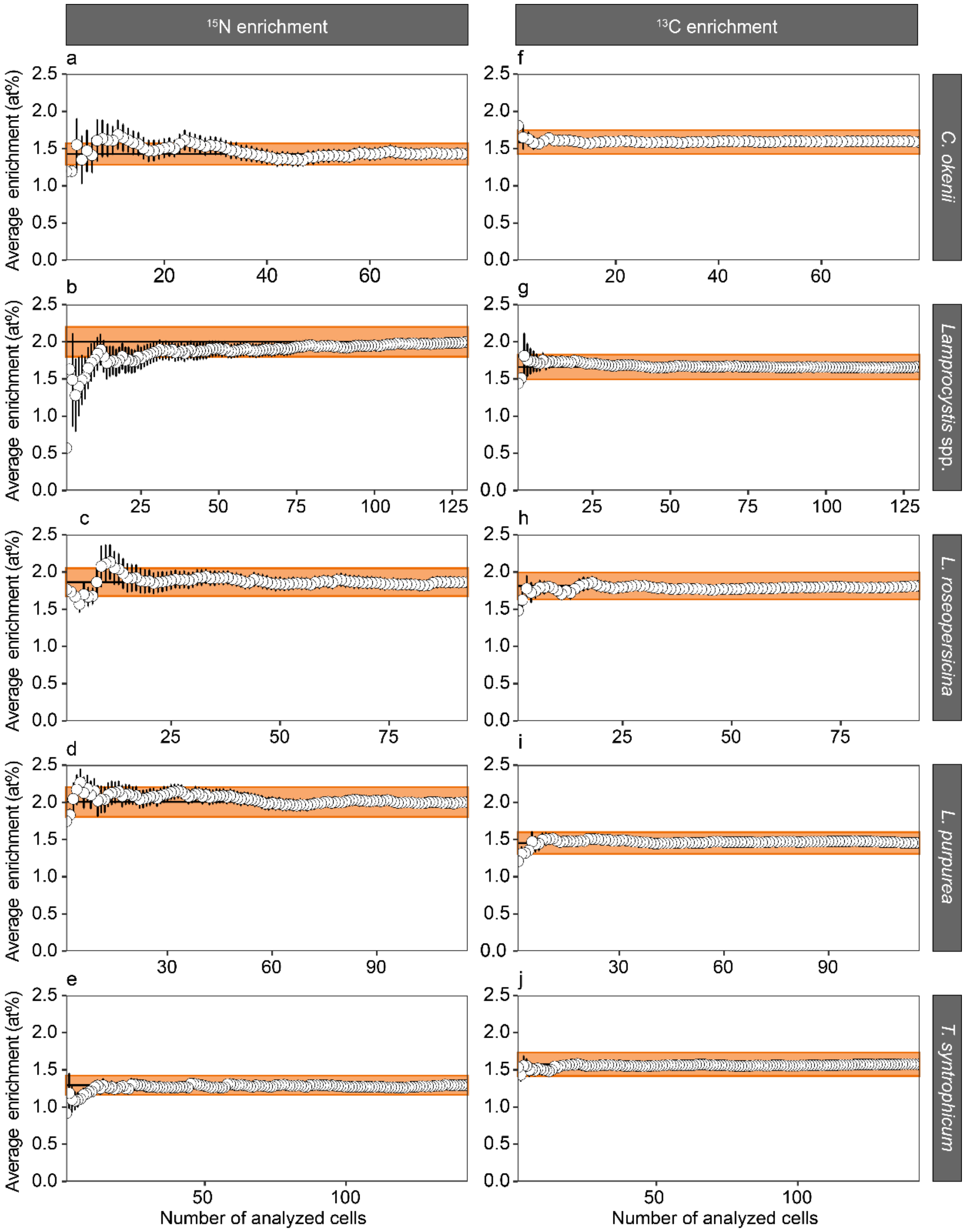


**Figure S10. The variability of $^{15}N$ at% (a-e) and $^{13}C$ at% (f-j) across measured cells.** Black lines represent the total mean with the orange shading indicating ≤10% deviation from the mean. The measured cells were ordered randomly and the mean enrichment was calculated with increasing number of regarded cells (white dots). The error bars represent the standard error of the mean enrichment. The plots were created in accordance with Svedén et al.[7].

# Supplementary References


1. Luedin SM*, et al.* Draft Genome Sequence of *Chromatium okenii* Isolated from the Stratified Alpine Lake Cadagno. *Scientific Reports* **9**, 1936 (2019).

2. Berg JS*, et al.* Dark aerobic sulfide oxidation by anoxygenic phototrophs in anoxic waters. *Environmental Microbiology* **21**, 1611-1626 (2019).

3. Dos Santos PC, Fang Z, Mason SW, Setubal JC, Dixon R. Distribution of nitrogen fixation and nitrogenase-like sequences amongst microbial genomes. *BMC Genomics* **13**, 162 (2012).

4. Halm H*, et al.* Co-occurrence of denitrification and nitrogen fixation in a meromictic lake, Lake Cadagno (Switzerland). *Environmental Microbiology* **11**, 1945-1958 (2009).

5. Mooshammer M*, et al.* Flow-through stable isotope probing (Flow-SIP) minimizes cross-feeding in complex microbial communities. *The ISME Journal*, (2020).

6. Tonolla M, Demarta A, Peduzzi R, Hahn D. In Situ Analysis of Phototrophic Sulfur Bacteria in the Chemocline of Meromictic Lake Cadagno (Switzerland). *Applied and Environmental Microbiology* **65**, 1325-1330 (1999).

7. Svedén JB*, et al.* High cell-specific rates of nitrogen and carbon fixation by the cyanobacterium *Aphanizomenon* sp. at low temperatures in the Baltic Sea. *FEMS Microbiology Ecology* **91**, (2015).
