# Supplementary material for: Purple sulfur bacteria fix N2 via molybdenum-nitrogenase in a low molybdenum Proterozoic ocean analogue
Source: Nat Commun. 2021 Aug 6;12:4774. doi: 10.1038/s41467-021-25000-z (PMC8346585; doi:10.1038/s41467-021-25000-z)
Supplement: Supplementary file 4 — Description of additional supplementary files [file 41467_2021_25000_MOESM4_ESM.docx]

Description of additional supplementary information

Title; supplementary dataset 1

Description: cell counts, fixation rates and detection limits, NifH, NifD and NifK tree sequences and detailed MAG information.
