# Supplementary material for: Purple sulfur bacteria fix N2 via molybdenum-nitrogenase in a low molybdenum Proterozoic ocean analogue
Source: Nat Commun. 2021 Aug 6;12:4774. doi: 10.1038/s41467-021-25000-z (PMC8346585; doi:10.1038/s41467-021-25000-z)
Supplement: Supplementary file 5 — Reporting Summary [file 41467_2021_25000_MOESM5_ESM.pdf]

nature portfolio

Corresponding author(s): Katharina Kitzinger
Last updated by author(s): Jul 11, 2021

Reporting Summary

Nature Portfolio wishes to improve the reproducibility of the work that we publish. This form provides structure for consistency and transparency in reporting. For further information on Nature Portfolio policies, see our Editorial Policies and the Editorial Policy Checklist.

Statistics

For all statistical analyses, confirm that the following items are present in the figure legend, table legend, main text, or Methods section.

n/a	Confirmed	
☐	☒	The exact sample size (*n*) for each experimental group/condition, given as a discrete number and unit of measurement
☐	☒	A statement on whether measurements were taken from distinct samples or whether the same sample was measured repeatedly
☐	☒	The statistical test(s) used AND whether they are one- or two-sided<br>*Only common tests should be described solely by name; describe more complex techniques in the Methods section.*
☒	☐	A description of all covariates tested
☒	☐	A description of any assumptions or corrections, such as tests of normality and adjustment for multiple comparisons
☐	☒	A full description of the statistical parameters including central tendency (e.g. means) or other basic estimates (e.g. regression coefficient) AND variation (e.g. standard deviation) or associated estimates of uncertainty (e.g. confidence intervals)
☐	☒	For null hypothesis testing, the test statistic (e.g. *F*, *t*, *r*) with confidence intervals, effect sizes, degrees of freedom and *P* value noted<br>*Give P values as exact values whenever suitable.*
☒	☐	For Bayesian analysis, information on the choice of priors and Markov chain Monte Carlo settings
☒	☐	For hierarchical and complex designs, identification of the appropriate level for tests and full reporting of outcomes
☒	☐	Estimates of effect sizes (e.g. Cohen's *d*, Pearson's *r*), indicating how they were calculated

Our web collection on statistics for biologists contains articles on many of the points above.

Software and code

Policy information about availability of computer code

Data collection	No software was used for data collection.
Data analysis	Microsoft Excel 2016, BBDuk v37.24, BBMerge v37.24, metaSPAdes version 3.14.0, anvi'o version 6.2, Bowtie 2 version 2.3.5.1, samtools version 1.10, Prodigal V2.6.3, GhostKOALA version 2.2, Centrifuge version 1.0.4, blast, MetaBAT version 2.12.1, concoct version 1.1.0, DAS Tool version 1.1.2, CheckM v1.0.18, GTDB-Tk v1.1.0, featureCounts version v2.0.1, ROCker version 1.2.0, HMMER version 3.1b2, SortMeRNA version 4.2.0, phyloFlash v3.3b2, R version 3.6.1, MAFFT version 6.717b, RAxML version 8.2.12, Taxtastic v0.9.0, pplacer and guppy v1.1.alpha19-0-g807f6f3, iTOL v5, ARB version 6.1, Look@NanoSIMS 2015-10-20, Zeiss Zen blue software 3.2<br><br>The custom R function used for the correction of sampling depth is accessible via https://github.com/mirimarine/N2-fixation-in-Lake-Cadagno.

For manuscripts utilizing custom algorithms or software that are central to the research but not yet described in published literature, software must be made available to editors and reviewers. We strongly encourage code deposition in a community repository (e.g. GitHub). See the Nature Portfolio guidelines for submitting code & software for further information.

Data

Policy information about availability of data

All manuscripts must include a data availability statement. This statement should provide the following information, where applicable:

- Accession codes, unique identifiers, or web links for publicly available datasets
- A description of any restrictions on data availability
- For clinical datasets or third party data, please ensure that the statement adheres to our policy

The sequence data generated in this study is deposited in the NCBI database. Metatranscriptomic data is deposited under BioProject number PRJNA693537 and

BioSample numbers SAMN17390591, SAMN17390592 and SAMN17390593. Nif-gene encoding MAGs, generated from metagenome data retrieved from BioProject PRJEB22995, are deposited under BioProject PRJNA697932 and BioSample numbers SAMN17492688 to SAMN17492723. Cell counts, fixation rates and detection limits, NifH, NifD and NifK tree sequences and detailed MAG information are available in Supplementary File 1. Source data are provided with this paper. The databases NCBI SRA, NCBI non-redundant protein sequences (nr) and SILVA 138 SSU were used for data analyses.

Field-specific reporting

Please select the one below that is the best fit for your research. If you are not sure, read the appropriate sections before making your selection.

☐ Life sciences ☐ Behavioural & social sciences ☒ Ecological, evolutionary & environmental sciences

For a reference copy of the document with all sections, see nature.com/documents/nr-reporting-summary-flat.pdf

Ecological, evolutionary & environmental sciences study design

All studies must disclose on these points even when the disclosure is negative.

Study description	We performed incubations with stable isotopes to determine water column N2 and CO2 fixation rates using water from the Lake Cadagno chemocline in 2018. Incubations were performed at three (August 28th) or one (August 29th) selected depth(s) in independent biological triplicates each. Sampling depths were selected based on the in situ CTD profile. For the incubated sample from August 28th with the highest bulk N2 fixation rate, we performed nanoSIMS analysis to determine single cell N2 and CO2 fixation rates. We investigated five distinct purple sulfur bacterial populations, with ≥79 cells analyzed per population. We also analyzed one purple sulfur bacteria population (Chromatium okenii) using nanoSIMS in an additional sample obtained and incubated on August 29th as independent replicate experiment. Additionally, we analyzed metagenomic data previously obtained from Lake Cadagno in 2014 (Berg et al. 2019, doi.org/10.1111/1462-2920.14543), and obtained metatranscriptomes from 2018 samples which were analyzed for nitrogenase encoding gene presence and transcription, as well as overall microbial activity by 16S rRNA gene transcription levels.
Research sample	Bulk N2 and CO2 fixation rates by the bulk microbial community were examined at three depths in and below the chemocline of Lake Cadagno. Depths selection was based on previous studies reporting N2 fixation activity in the chemocline, which also harbors the highest cell densities in Lake Cadagno. For nanoSIMS analysis, we picked 5 purple sulfur bacterial populations based on their genetic potential for N2 fixation, their active transcription of N2 fixation related genes at the time of sampling and their high activity and abundance. The investigated purple sulfur bacteria populations were Chromatium okenii, Thiodictyon syntrophicum, Lamprocystis purpurea, Lamprocystis roseopersicina and Lamprocystis spp..
Sampling strategy	No statistical methods were used to pre-determine sample-size. Stable isotope incubations to determine N2 and CO2 fixation rates were performed in three independent biological replicates per depth, which is standard procedure for environmental biogeochemical rate measurements (e.g. Großkopf et al. 2012, https://doi.org/10.1038/nature11338; Bonnet et al. 2013, https://doi.org/10.1371/journal.pone.0081265; Martínez-Pérez et al. 2016, https://doi.org/10.1038/nmicrobiol.2016.163). We performed statistical tests to ensure that sufficient cells were analyzed with nanoSIMS according to Svedén et al. 2015 (https://doi.org/10.1093/femsec/fiv131).
Data collection	Field sampling was performed by M. Philippi, J. S. Berg, M. M. M. Kuypers, C. J. Schubert and N. Storelli. In situ water profiles were measured using a CTD system and recorded with an attached laptop. Water sampling, incubation set up, water treatment and filtration details were recorded in a labbook. NanoSIMS measurements were performed by K. Kitzinger, S. Littmann and A. T. Kidane, measurements details were noted and data was saved on multiple hard drives connected to the machine.
Timing and spatial scale	Sampling was done during the productive summer season, where Lake Cadagno is snow- and ice-free and accessible for sampling. Sampling was done on the 28th and 29th of August 2018 at the deepest part of Lake Cadagno (ca. 21m depth) and included one CTD cast per day and 20 water sampling depths around the chemocline (12-17m depth). Incubation experiments with stable isotopes from 3 selected depths (13.7 m, 14 m, 15.5 m) were performed on 28th August and 1 depth (13.5 m) on 29th of August on site and started on the day of sampling. Incubation experiments were terminated on site, 24 hours after incubation start.
Data exclusions	No data was excluded from the study. N2 and CO2 fixation rates from 15.5 m depth (28.9.2018) are available only in duplicates, as one replicated was lost during the incubation (incubation bottle broke).
Reproducibility	All attempts at replication were successful. Similar results were obtained for all independent biological replicates. Data was analyzed by at least two different people independently.
Randomization	Incubation bottles were placed randomly inside the incubator setup. Mass spectrometry samples were measured in randomized sequence and identified through a code.
Blinding	The person performing the mass spectrometry measurements had no knowledge of the sample identity (samples were identified via their sampling code).

Did the study involve field work? ☒ Yes ☐ No

Field work, collection and transport

Field conditions	Sampling at Lake Cadagno was performed in late summer of 2018 (28th and 29th of August). The weather was mild and sunny. Lake surface water had a temperature of ~16°C, bottom water had a temperature of ~4°C.

Location	Lake Cadagno, Switzerland. 46.550278 N 8.713333 E. 1,920 m surface elevation. Sampling was performed at the deepest part of the Lake (21 m). Water samples were collected from ca. 12 to 17 m water depth.
Access & import/export	Access was provided by the Alpine Biology Center Foundation (Switzerland) who granted us permission to use their research facility at Lake Cadagno (billing nr. 212018; Bellinzona, 12.09.2018). Project collaborators from the Eawag (Swiss Federal Institute of Aquatic Science and Technology) and the University of Applied Sciences of Southern Switzerland (SUPSI) provided logistics and sampling equipment.
Disturbance	To reduce ecosystem disturbance, sampling and laboratory work was restricted to the lake's sampling platform and the research center of the Alpine Biology Center Foundation, which are frequently used for scientific and educational purposes.

Reporting for specific materials, systems and methods

We require information from authors about some types of materials, experimental systems and methods used in many studies. Here, indicate whether each material, system or method listed is relevant to your study. If you are not sure if a list item applies to your research, read the appropriate section before selecting a response.

Materials & experimental systems

n/a	Involved in the study
☒	☐ Antibodies
☒	☐ Eukaryotic cell lines
☒	☐ Palaeontology and archaeology
☒	☐ Animals and other organisms
☒	☐ Human research participants
☒	☐ Clinical data
☒	☐ Dual use research of concern

Methods

n/a	Involved in the study
☒	☐ ChIP-seq
☒	☐ Flow cytometry
☒	☐ MRI-based neuroimaging
